# Acellularization-Induced Changes in Tensile Properties Are Organ Specific - An *In-Vitro* Mechanical and Structural Analysis of Porcine Soft Tissues

**DOI:** 10.1371/journal.pone.0151223

**Published:** 2016-03-09

**Authors:** Stefan Schleifenbaum, Torsten Prietzel, Gabriela Aust, Andreas Boldt, Sebastian Fritsch, Isabel Keil, Holger Koch, Robert Möbius, Holger A. Scheidt, Martin F. X. Wagner, Niels Hammer

**Affiliations:** 1 Department of Orthopedic, Trauma and Plastic Surgery, University of Leipzig, Germany; 2 Department of Surgery, Research Laboratories, University of Leipzig, Germany; 3 Institute of Clinical Immunology, University of Leipzig, Germany; 4 Institute of Materials Science and Engineering, Technische Universität Chemnitz, Chemnitz, Germany; 5 Institute of Anatomy, University of Leipzig, Leipzig, Germany; 6 Translational Centre for Regenerative Medicine (TRM), Leipzig, Germany; 7 Institute for Medical Physics and Biophysics, University of Leipzig, Leipzig, Germany; 8 Department of Anatomy, University of Otago, Dunedin, New Zealand; University of California, San Diego, UNITED STATES

## Abstract

**Introduction:**

Though xenogeneic acellular scaffolds are frequently used for surgical reconstruction, knowledge of their mechanical properties is lacking. This study compared the mechanical, histological and ultrastructural properties of various native and acellular specimens.

**Materials and Methods:**

Porcine esophagi, ureters and skin were tested mechanically in a native or acellular condition, focusing on the elastic modulus, ultimate tensile stress and maximum strain. The testing protocol for soft tissues was standardized, including the adaption of the tissue’s water content and partial plastination to minimize material slippage as well as templates for normed sample dimensions and precise cross-section measurements. The native and acellular tissues were compared at the microscopic and ultrastructural level with a focus on type I collagens.

**Results:**

Increased elastic modulus and ultimate tensile stress values were quantified in acellular esophagi and ureters compared to the native condition. In contrast, these values were strongly decreased in the skin after acellularization. Acellularization-related decreases in maximum strain were found in all tissues. Type I collagens were well-preserved in these samples; however, clotting and a loss of cross-linking type I collagens was observed ultrastructurally. Elastins and fibronectins were preserved in the esophagi and ureters. A loss of the epidermal layer and decreased fibronectin content was present in the skin.

**Discussion:**

Acellularization induces changes in the tensile properties of soft tissues. Some of these changes appear to be organ specific. Loss of cross-linking type I collagen may indicate increased mechanical strength due to decreasing transverse forces acting upon the scaffolds, whereas fibronectin loss may be related to decreased load-bearing capacity. Potentially, the alterations in tissue mechanics are linked to organ function and to the interplay of cells and the extracellular matrix, which is different in hollow organs when compared to skin.

## Introduction

Surgical reconstruction following soft tissue injuries includes a variety of techniques which also use materials of biological origin [1,2]. Some issues are related to the application of biological tissues, including their availability—especially if these tissues originate from human donors. Another important aspect is the antigenicity induced by the donor tissue. One approach to solve this issue is to use hetero- or xenogeneic tissues further processed to acellular scaffolds [3–8]. Acellular scaffolds have reduced antigenicity [9] and provide a matrix exclusively consisting of extracellular matrix (ECM), which likely facilitates the processes of healing and biointegration [10].

Acellular scaffolds are frequently used in cardiac and vascular surgery [11–13], and in soft tissue repair of muscles, tendons and ligaments [14–17]. Also, scaffolds are used for plastic surgery following nerve injury [18] and tumors of the genitourinary tract [19–21]. In other studies, the biointegration processes of acellular implants were determined [7,21,22]. However, though the scaffolds are frequently applied, there is a lack of data characterizing them mechanically and comparing the acellular to the native condition, which may to some extent reflect tissue mechanics after integration into the host. Given this lack of mechanical data examined porcine esophagus, ureter and skin samples mechanically in both the native and the acellular condition, complementing histological and *in-vivo* studies of Koch et al. [21,22].

In previous studies we established and optimized a protocol to standardize the mechanical testing procedure of soft tissues, including partial plastination of the samples’ ends [23–26] and adjustment of the samples’ water content [25]. In the given study, we further optimized the protocol concerning sample dimensions. This approach aimed at easing the identification of the failure location. We applied this protocol to obtain mechanical properties of esophagus, ureter and skin samples and to compare these data at the microscopic and ultrastructural level. The following hypotheses were addressed:

1. Tensile properties of native or acellular porcine samples are different in esophagi, ureters and skin.
2. Acellularization causes changes in the tensile properties of esophagi, ureters and skin, as compared to the native condition.
3. Changes in the tensile behavior of acellular scaffolds are accompanied by morphological alterations in the scaffolds.

## Materials and Methods

**Fig 1:**
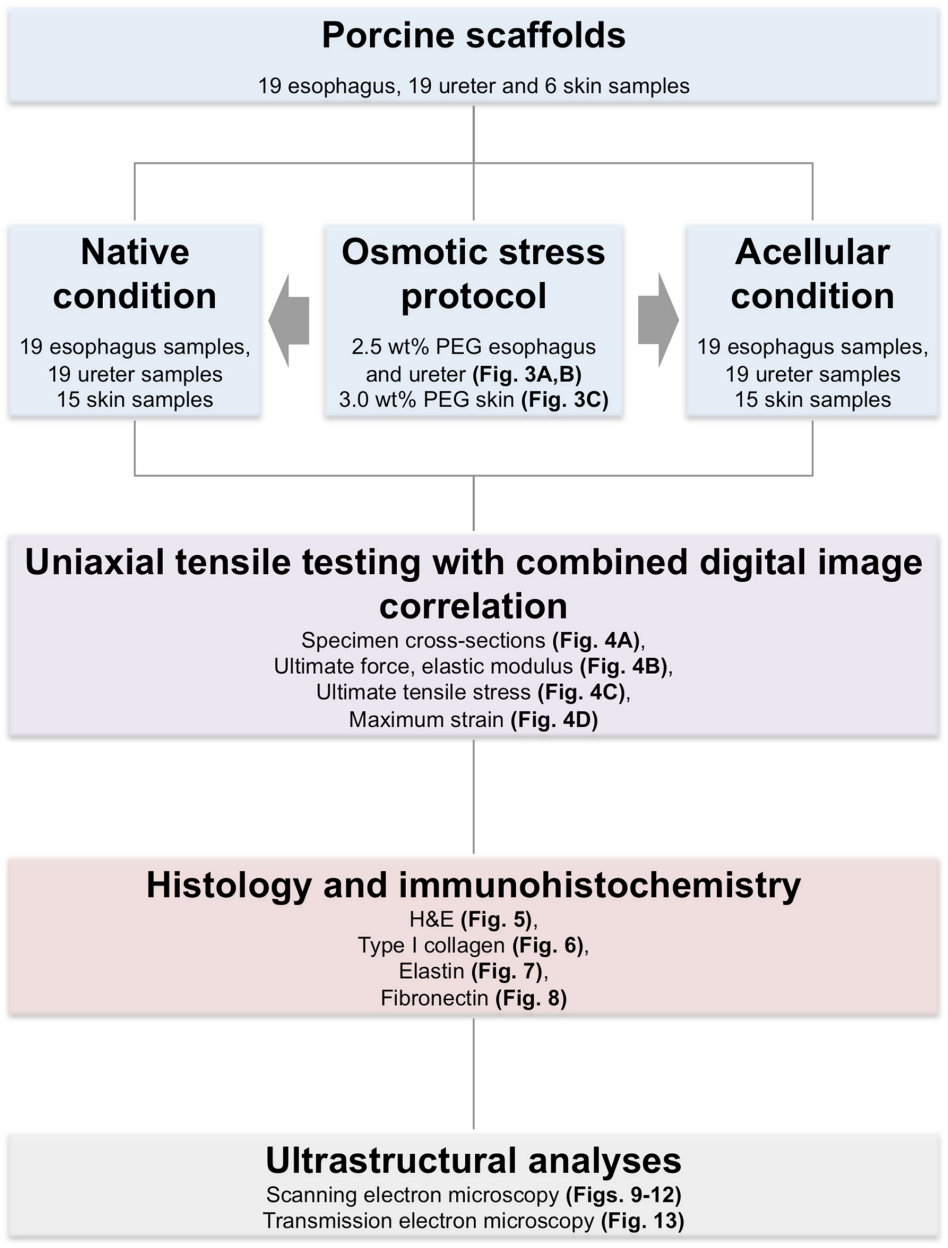
Flow chart summarizing the experimental protocol.

The tissues were obtained from four to seven month-old pigs post mortem following experiments approved by the Landesdirektion Sachsen animal welfare committee (TVV 38/12). Each specimen was prepared in a native and anatomically unfixed condition under constant moistening, precooled at 3°C and subsequently shock frozen at -85°C. The specimens were obtained within 24 hours or less to minimize potential effects of autolysis. 19 esophagi, 19 ureters and six skin specimens were obtained. A graphical summary of the experimental protocol is depicted in Fig 1.

**Mechanical testing group:** The esophagus and ureter specimens were further subdivided lengthwise cranial-caudally into two samples, resulting in 19 matched pairs of 2 samples each. The skin specimens were divided in 30 samples. One of each pair of samples were subjected to the acellularization procedure. The native counterparts remained frozen.

**Osmotic stress group:** Additionally, 258 small-scale samples were obtained from the contra-lateral side of each of the same individuals for determining the water content and establishing an osmotic stress protocol.

### Acellularization procedure

The acellularization procedure was carried out according to [21,22]. To remove the cellular components the specimens were submerged in sodium dodecyl sulphate (SDS) for seven days in case of the esophagi and ureters and for 28 days in case of the skin specimens. Following this, the samples were rinsed 7 days in distilled water and shock frozen.

### Osmotic stress protocol for adjusting the water content of the samples

The osmotic stress technique was applied as shown previously. [25]. Small parts of the native specimens of each tissue were used to determine the water content in the native condition. Seven native and seven acellular samples each were submerged in a tris-phosphate-buffered polyethylengylcol (PEG) solution at concentrations of 2.0, 2.5 and 3.0 wt-% for 1, 2, 4, 8, 11 and 24 hours. Each specimen dialysed (Carl Roth GmbH + Co. KG, Karlsruhe, Germany; molecular weight cut off = 6–8 kDa). Afterwards, their water content was determined using lyophilization.

### Sample plastination and mechanical testing

Both ends of the samples of the mechanical testing group were partially plastinated [23,25,26]. The central native or acellular parts of the samples were mounted between two templates to standardize their initial measurement sample length to 50 mm. Following this, the samples’ water content was osmotically adapted for 24 hours to the respective native value of each tissue. For this purpose, 2.5-wt% PEG solutions were used for the esophagi and ureters and three-wt% PEG solutions for the skin.

**Fig 2:**
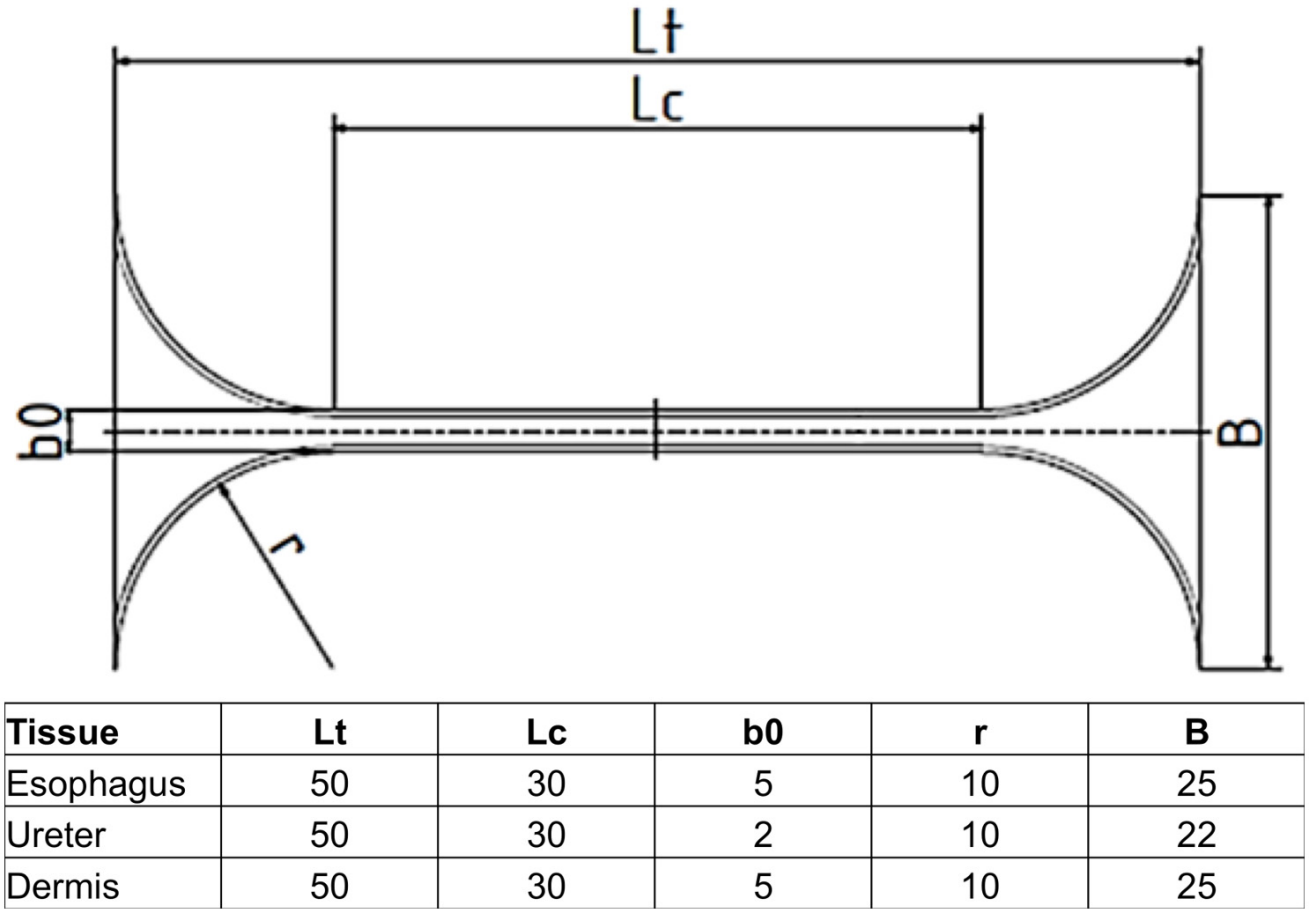
Template adapted from the DIN 50125 with standardized dimensions for the esophagus, ureter and skin samples.

For standardized tensile testing, the samples’ width was sectioned using a template adapted from the German standard DIN 50125 [27]. The geometry of the template was adapted to the width of the three tissues as shown in Fig 2. The samples were clamped in a uniaxial testing machine (Typ 5566A, Instron, Norwood, MA, USA) and ten preconditioning cycles were applied to each sample with the following parameters: crosshead velocity v = 20 mm/min, minimum and maximum strain 0% to 5% of the initial length. In a next step, the cross-sectional areas were casted (VPS Hydro 380, Henry Schein Medical GmbH, Hamburg, Germany and REF 2112, Voco GmbH, Cuxhaven, Germany). Following this, a speckle pattern was sprayed onto each sample for digital image correlation. Then the tensile tests were performed with v = 20 mm/min and a strain corresponding to material failure, as indicated by a force-level decrease of at least 30% of the respective maximum value. The local stress-stain data were recorded using a digital image correlation system (Limess Meßtechnik und Software GmbH, Krefeld, Germany). The sample data were only evaluated from the respective experiment if they met all of the following inclusion criteria:

1. Material failure inside the region of parallel measuring length.
2. No macroscopically visible material slippage.
3. Sufficient data from digital image correlation for the determination of stress-strain-curves.

### Histology and immunohistochemistry

The samples from the mechanical testing group with the template were fixed in paraformaldehyde, paraffin-embedded and sectioned at 5 μm (Fig 1). Hematoxylin-eosin (HE) staining was carried out as reported previously [25]. For immunohistochemical staining, deparaffinized tissue sections were incubated with 1:100-diluted collagen I, elastin (both Acris Antibodies, Herford, Germany) or fibronectin (Dianova, Berlin, Germany) antibodies. Primary antibody binding was detected with the Envision DAB kit (DAKO Deutschland GmbH, Hamburg, Germany).

### Ultrastructural analyses

Tissue samples of 5 to 10 mm^3^ were prepared from native and acellular esophagus and ureters (both from the muscularis propria) and from the skin samples (subepidermal region), as indicated in Fig 1. The samples were immediately fixed in 3% glutaraldehyde/0.1 M phosphate buffer (pH = 7.4) and then washed in distilled water. The samples used for scanning electron microscopy (SEM) were dehydrated, followed by the critical point drying process (CPD-030, Bal-Tec AG, Liechtenstein). SEM was carried out using a JSM-6700F field emission microscope (JEOL Ltd., Tokyo, Japan) at magnifications between 1000x and 50,000x.

The samples for transmission electron microscopy (TEM) were washed in 0.1 M cacodylate buffer, post-fixed in 1% osmium tetroxide for 1 hour, again washed in distilled water, and dehydrated and infiltrated in ascending ethanol series, propylene oxide and resin. Semi-thin sections (1 μm) were used for orientation before ultrathin sections were cut and collected on copper slot grits. The samples were contrasted with uranyl acetate and lead citrate. A Philips CM100 BioTWIN (Philips/FEI Corporation, Eindhoven, The Netherlands) equipped with a MegaView III digital camera (Olympus Soft Imaging Solutions GmbH, Münster, Germany) was used to obtain TEM images at magnifications between 9700x and 66,000x.

### Data processing and statistical analysis

The casts of the cross-sections were scanned with a resolution of 1200 dpi (Perfection 7V750Pro, Seiko Epson Corporation, Suwa, Japan) and calculated using Measure 2.1d (DatInf GmbH, Tübingen, Germany). To determine the local stress and strain data during mechanical testing, the ISTRA 4D software (VRS 4.4.1.354, Dantec Dynamics, Ulm, Germany) was used. Elastic modulus, ultimate tensile stress and maximum strain (strain at ultimate tensile stress) was calculated using MATLAB 2011 (Mathworks, Natick, MA, USA). SPSS 20.0 software (IBM, IL, USA) and Excel 2013 (Microsoft Corporation, Redmond, WA, USA) were used to evaluate the data. Following the Kolmogorov-Smirnov test to determine normal distribution, comparison of the native to the acellular condition of the specimens was performed with the Wilcoxon-Signed-Ranks tested or the paired Student's T-test. For a comparison between the different tissues, the ANOVA test with post-hoc analyses was applied. *P* values of 5% or less were considered as statistically significant. All values were presented in mean values ± standard deviations.

## Results

### Native water content was different for esophagus, ureter and skin

**Fig 3:**
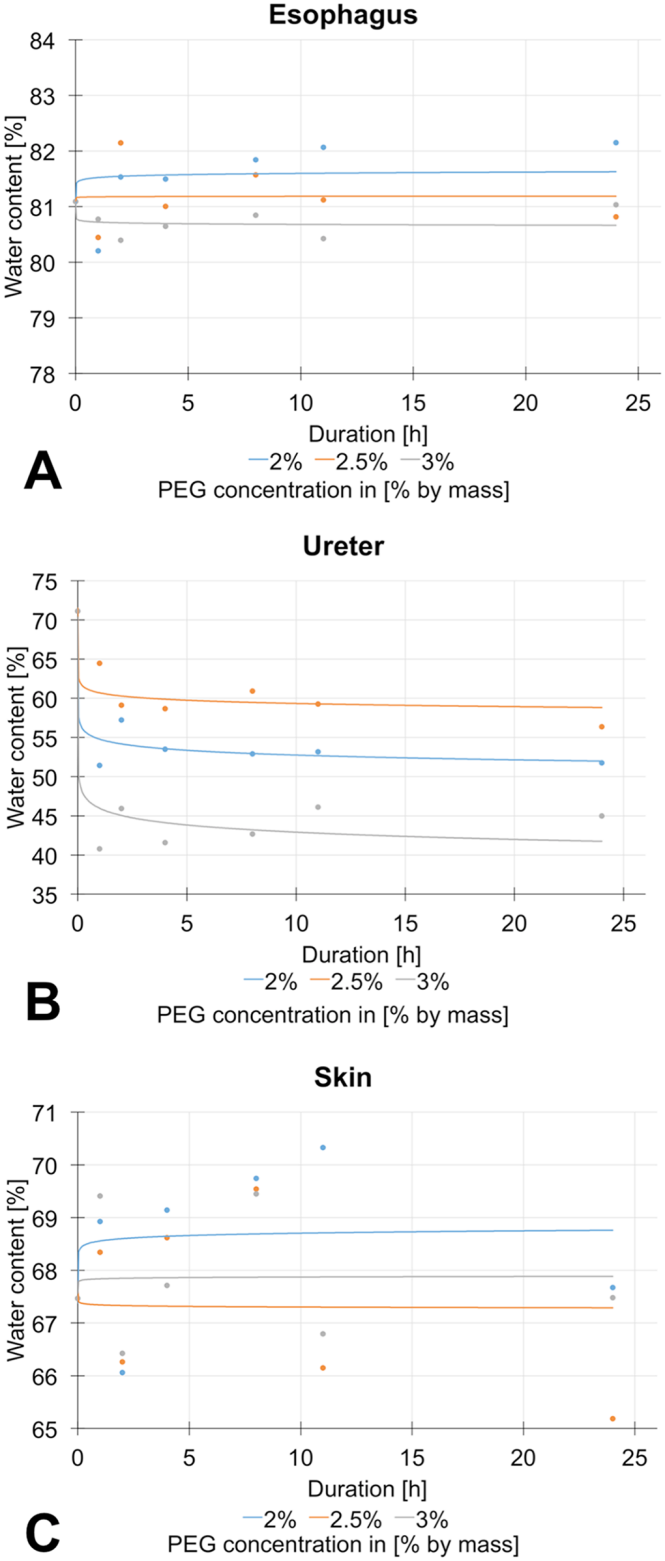
Osmotic stress protocol with different polyethylengylcol (PEG) concentrations for 3A) esophagus 3B) ureter 3C) skin samples.

The initial water content of the tissues was: 80.8 ± 0.9% for the esophagus, 56.4 ± 7.3% for the ureter and 67.5 ± 3.1% for the skin. In order to adjust the water content of the native and the acellular samples to an average value in the native condition, 2.5-wt% PEG solutions were used for the ureters and esophagi, whereas 3.0-wt% were used for the skin. The osmotic protocols are given in Fig 3.

### Evaluation of esophagus, ureter and skin tensile data

Stress-strain data of 10/19 native and 8/19 acellular esophagi, 12/19 native and 14/19 acellular ureters were considered valid. The excluded samples failed to meet a speckle pattern sufficient for strain analysis throughout testing. 7/15 native and 10/15 acellular skin samples were considered valid. In the excluded skin samples, material slippage occurred and/or an insufficient speckle pattern hampered data acquisition.

### Cross sections decreased to a different extent after acellularization

**Fig 4:**
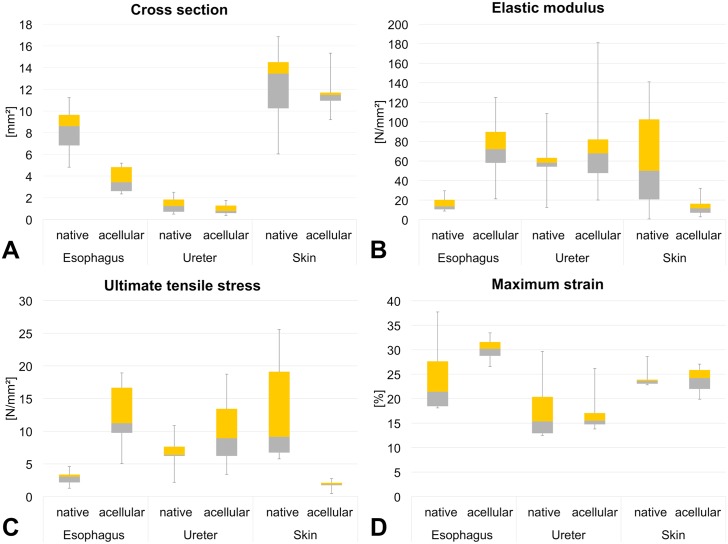
Boxplot from esophagus, ureter and skin samples in the native and acellular condition 4A) cross section 4B) elastic modulus 4C) ultimate tensile stress 4D) maximum strain.

The cross sections of the native esophagi were significantly higher than those of the acelluar esophagi with mean values of 8.2 ± 2.0 mm^2^ and 3.7 ± 1.1 mm^2^ (p = 0.002; Fig 4; Table 1), respectively. The ureters showed the same trend on a non-significant level (p = 0.111) with 1.3 ± 0.7 mm^2^ in the native condition and 0.9 ± 0.4 mm^2^ in the acellular condition. The behavior of the skin was similar with 12.4 ± 3.3 mm^2^ in the native condition and 11.7 ± 1.6 mm^2^ in the acellular condition.

**Table 1:** Mean and standard deviation with the *p* value for the comparison native versus acellular condition.

	Cross section [mm²]		Elastic modulus [N/mm²]		Maximum strain [%]		Ultimate tensile stress [N/mm²]	
	MW	SD	MW	SD	MW	SD	MW	SD
**Esophagus**								
** native**	8.23	0.66	16.1	7.08	24.65	7.93	2.9	0.99
** acellular**	3.67	0.44	73.22	30.89	30.09	2.49	12.38	4.51
***p value***	*0*.*002*		*0*.*002*		*0*.*104*		*<0*.*001*	
**Ureter**								
** native**	1.34	0.66	61.46	24.2	17.24	5.29	6.91	2.15
** acellular**	0.91	0.44	73.21	40.46	16.98	3.87	9.52	4.29
***p value***	*0*.*111*		*0*.*517*		*0*.*882*		*0*.*255*	
**Skin**								
** native**	12.41	3.29	62.56	52.26	24.24	1.99	13.16	7.36
** acellular**	11.73	1.57	12.83	7.87	23.83	2.53	1.84	0.55
***p value***	*0*.*484*		*0*.*052*		*0*.*841*		*0*.*009*	

### Ultimate force increased in acellular esophagus samples but decreased in skin samples

Ultimate force values were 23.9 ± 2.0 N for the native esophagi and 45.5 ± 5.0 N for the acellular esophagi. The ureters showed no change with 9.3 ± 1.4 N in the native and 8.7 ± 1.9 N in the acellular condition. In contrast, the skin samples were more resilient in the native than in the acellular condition with ultimate forces of 163.3 ± 24.2 N and 21.6 ± 0.9 N, respectively.

### Increased elastic moduli were observed in acellular esophagi and ureters but decreasing values were found in acellular skin samples

The esophagus specimens were significantly less stiff in the native condition compared to the acellular, with mean elastic moduli of 16.1 ± 7.1 MPa and 73.2 ± 30.9 MPa, respectively (p = 0.002; Fig 4; Table 1). The elastic moduli of the ureter samples were similar in the native than in the acellular condition with 61.5 ± 24.2 MPa and 73.2 ± 40.5 MPa, respectively. The elastic modulus tended to be higher in the native than in the acellular skin samples with 62.6 ± 52.3 MPa and 12.8 ± 7.9 MPa, respectively (p = 0.052). Comparison of the elastic moduli between the native esophagi and ureters yielded significantly lower values in the esophagus compared to the ureter and skin samples (p ≤ 0.02; Table 2). In the acellular samples, the skin was significantly less stiff than the ureters and the skin (p ≤ 0.002).

**Table 2:** *P* value for the comparison between the different tissues.

*P value*	Ureter vs. esophagus	Ureter vs. skin	Esophagus vs. skin
**Elastic modulus**			
** native**	*0*.*008*	*1*	*0*.*02*
** acellular**	*1*	*<0*.*001*	*0*.*002*
**Maximum strain**			
** native**	*<0*.*001*	*0*.*022*	*0*.*157*
** acellular**	*<0*.*001*	*0*.*007*	*0*.*042*
**Ultimate tensile stress**			
** native**	*0*.*960*	*0*.*011*	*<0*.*001*
** acellular**	*0*.*316*	*<0*.*001*	*<0*.*001*

### Ultimate tensile stress values increased in acellular esophagi and ureters but strongly decreased in skin samples

In the esophagi, ultimate tensile stress increased significantly with 2.9 ± 1.0 MPa and 12.4 ± 4.5 MPa in the native compared to the acellular condition (p < 0.001, Fig 4; Table 1). In the ureters 6.9 ± 2.2 MPa and 9.5 ± 4.3 MPa were determined for the native and acellular samples, respectively. An extended decrease in ultimate tensile stress was observed in the skin samples with native values of 13.2 ± 7.4 MPa and acellular values of 1.8 ± 0.6 MPa (p = 0.009). Native skin yielded higher ultimate tensile stress values compared to the esophagi and ureters (p ≤ 0.011), whereas acellular skin yielded lower ultimate tensile stress values (p < 0.001) when compared to the esophagus and ureters (Table 2).

### Maximum strain differed between the tissues but not between the native and acellular condition

Slight and non-significant decreases of maximum strain were observed in esophagus and skin samples. In contrast, maximum strain values increased in the acellular ureters (Fig 4; Table 1). Comparison of the tissues in the native condition yielded lower values of the ureters compared to the esophagus and skin samples (p ≤ 0.022; Table 2). In the acellular condition, higher maximum strain values were found in the esophagus compared to the skin samples and in the skin compared to the ureter samples (p ≤ 0.042).

### Histology and immunohistochemistry indicated cell removal and intact ECM

**Fig 5:**
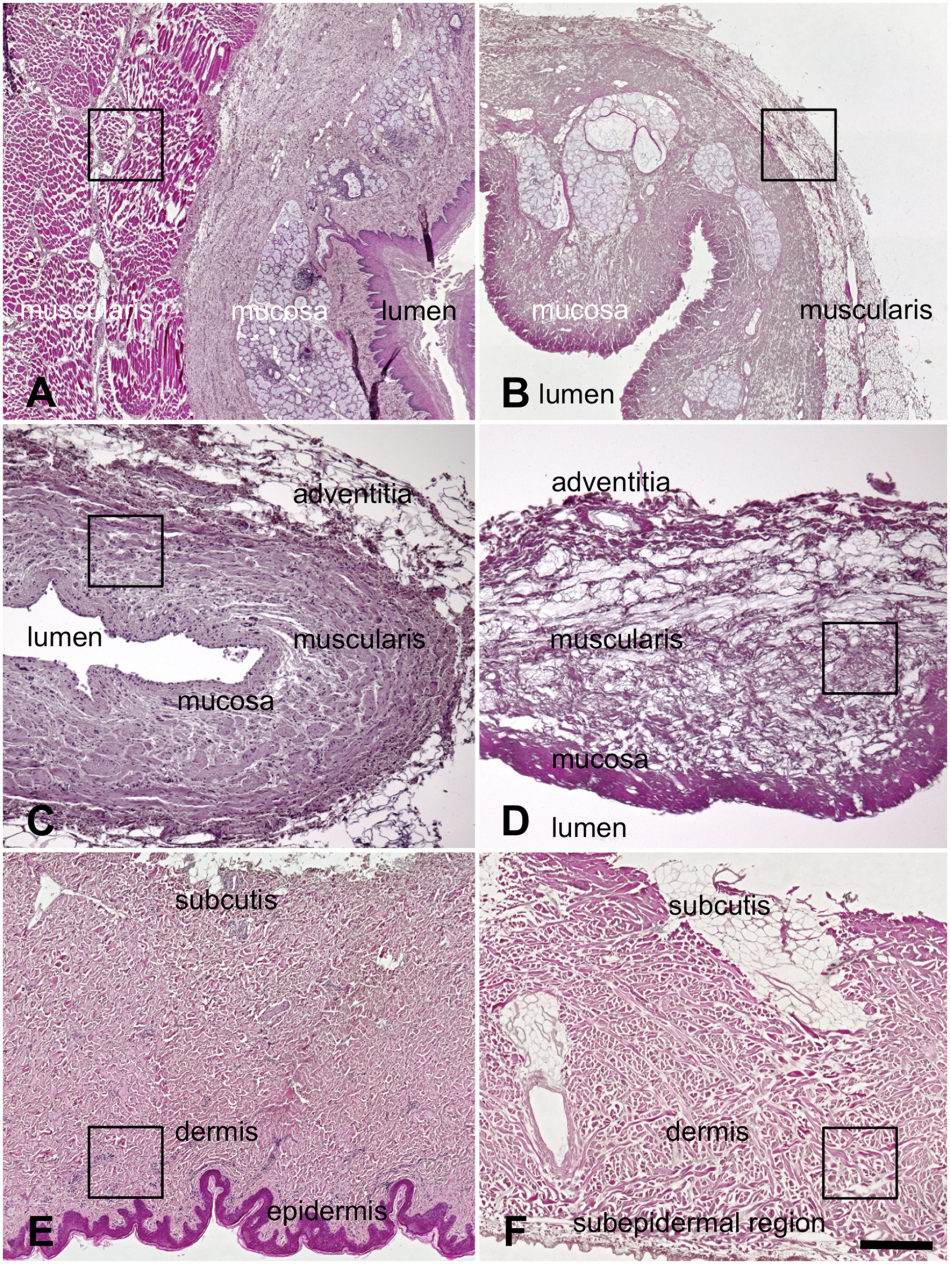
Hematoxylin-eosin stained-samples of 5A) native esophagus 5B) acellular esophagus 5C) native ureter 5D) acellular ureter 5E) native skin 5F) acellular skin. In spite of the removal of the cellular structures the respective tissue layers remained intact structurally. A marked thinning was observed in the tunica media of the esophagi and some thinning in the ureters. In the acellular skin samples the epidermis was completely removed. Black rectangles indicate the regions where samples for electron microscopy were obtained (also refer to Figs 9 to 13). Scale bar: 300 μm (5A,B), 100 μm (5C,D), 300 μm (5E,F).

In the HE-stained sections of all samples the tissue layers remained anatomically intact before and after acellularization. Distinct mucosal, submucosal and muscular layers were observed in the native esophagus (Fig 5A) and ureter samples (Fig 5C). The acellular esophagus (Fig 5B) and ureter samples (Fig 5D) showed wide gaps between the collagen matrix, especially in the tunica muscularis. The native skin samples showed a distinct stratum corneum, granulosum, mucosum and germinativum (Fig 5E). Cells were observed at varying numbers in the respective layers. The acellular skin samples showed no stratum corneum and no signs of cellular structures in the respective layers (Fig 5F). Thinning was observed in all samples following acellularization, being most pronounced in the esophagi.

**Fig 6:**
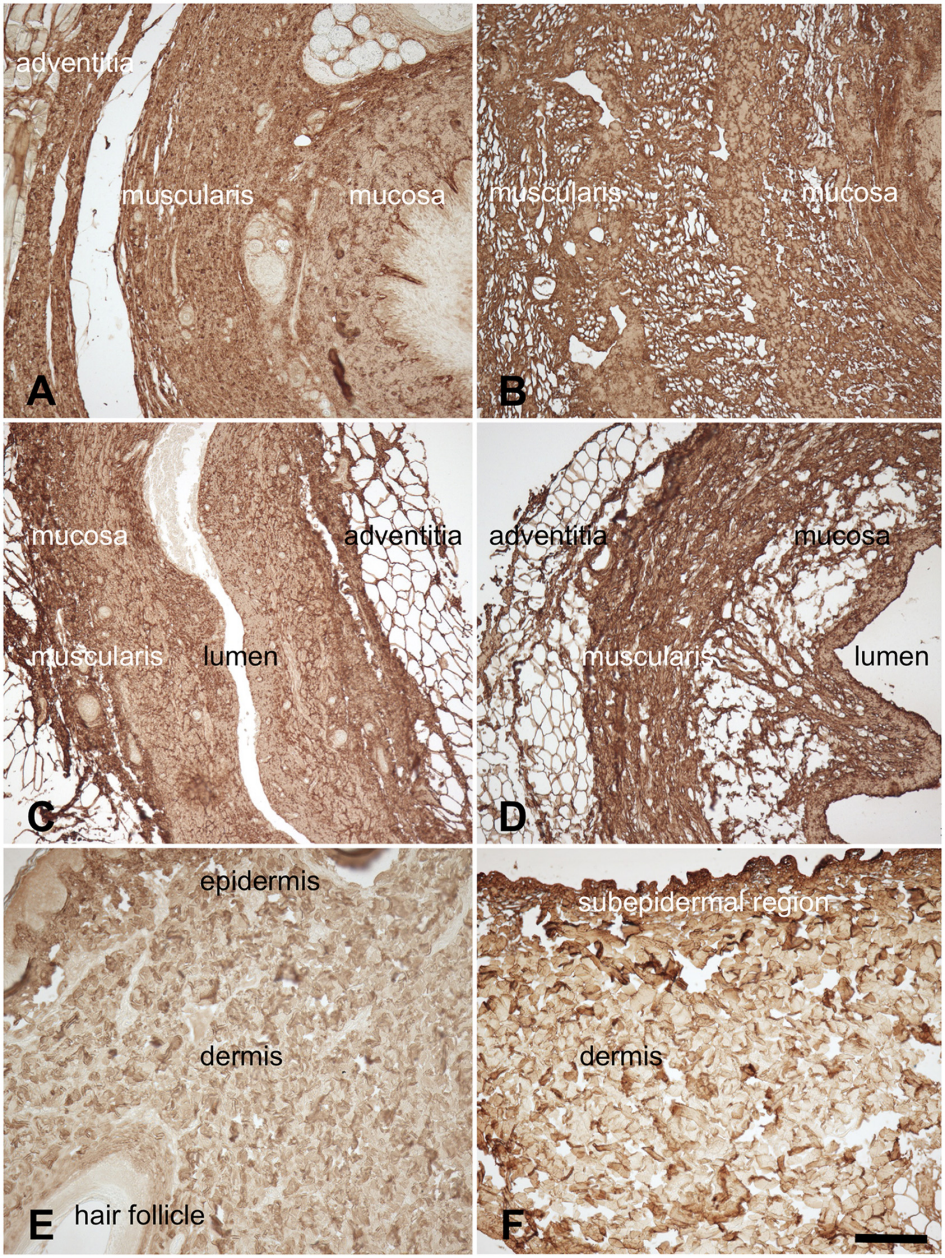
Anti-type I collagen staining of 6A) native esophagus 6B) acellular esophagus 6C) native ureter 6D) acellular ureter 6E) native skin 6F) acellular skin. Collagens were observed throughout the native and acellular scaffolds but appeared to be more condensed in the latter, especially in the tunica muscularis (6B,D) and in the subepidermal regions (6F). Scale bar 100 μm.

**Fig 7:**
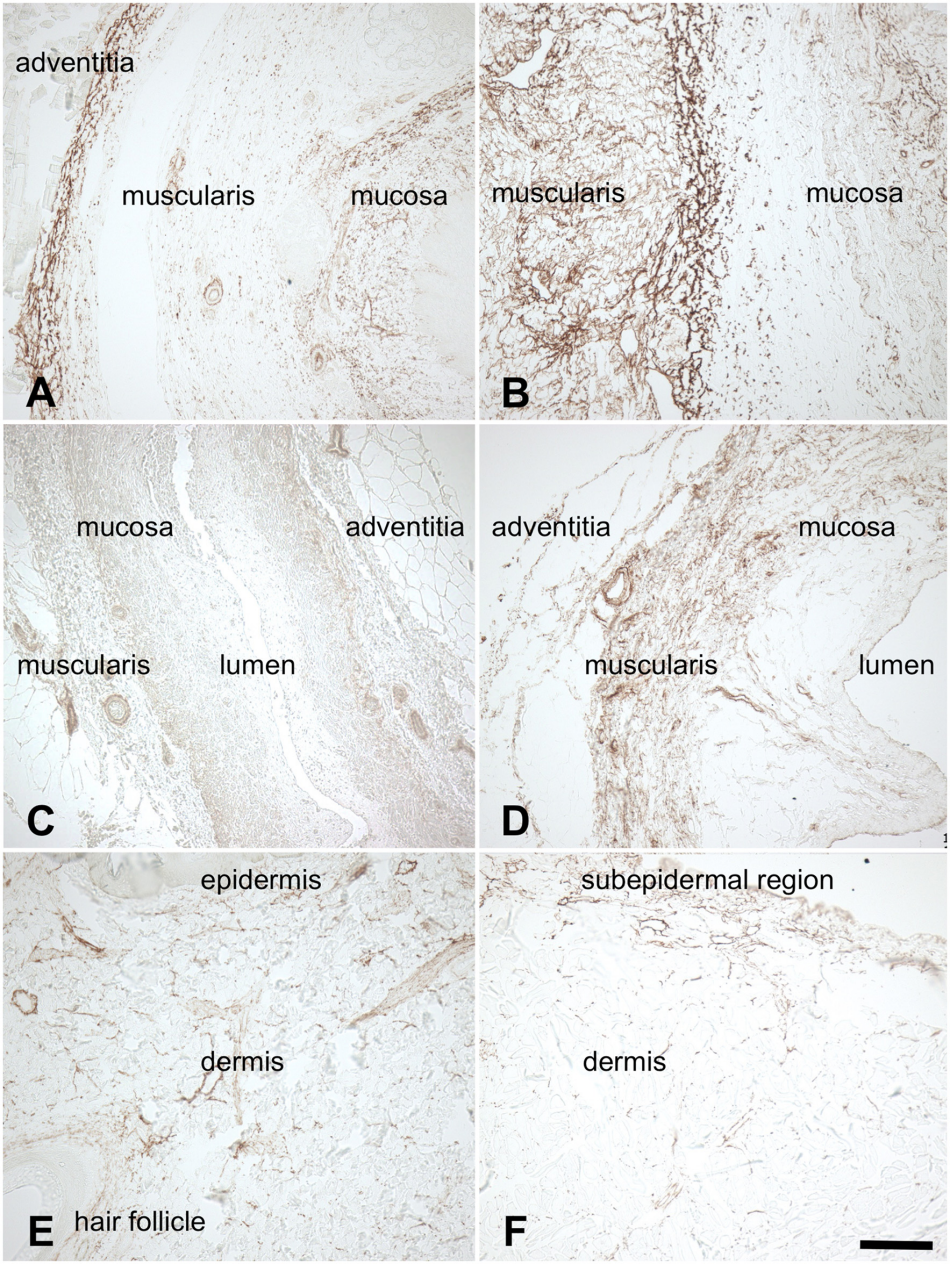
Anti-elastin staining of 7A) native esophagus 7B) acellular esophagus 7C) native ureter 7D) acellular ureter 7E) native skin 7F) acellular skin. Elastic fibers were observed in the submucosal regions, the tunica muscularis, to some extent in the adventitia and in blood vessels (7A-D). The skin samples showed low quantities of elastic fibers, mostly in the vessels and dermal perivascular regions (Fig 7E,F). Scale bar 100 μm.

**Fig 8:**
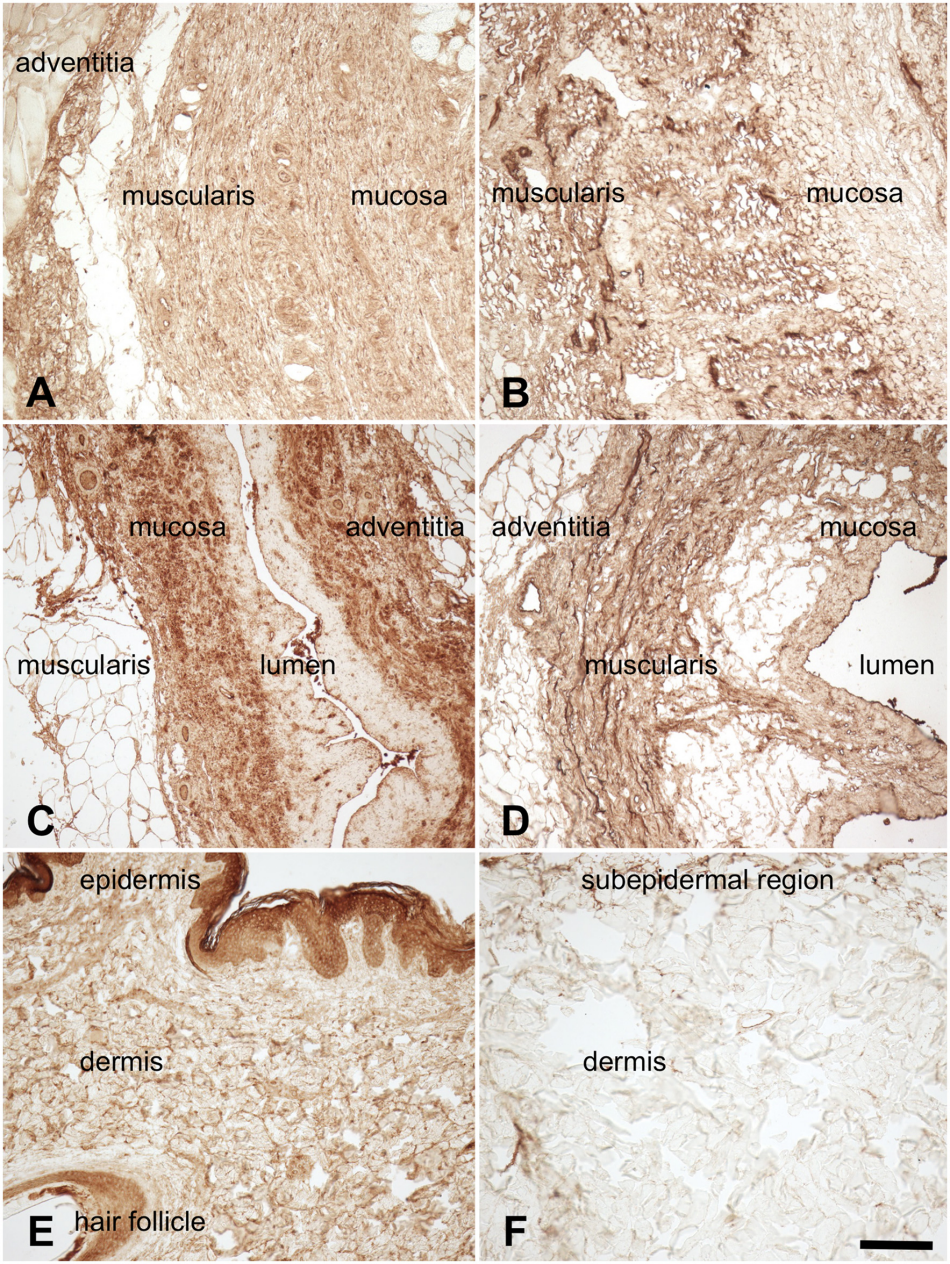
Anti-fibronectin staining of 8A) native esophagus 8B) acellular esophagus 8C) native ureter 8D) acellular ureter 8E) native skin 8F) acellular skin. Fibronectins were found throughout the ureters in the native (8A,C) and the acellular condition (8B,D), especially in the muscosal and muscular layers. A marked fibronectin washout was observed in the acellular (8F) compared to the native skin (8E) samples, especially in the dermal regions. Scale bar 100 μm.

Type I collagen was found throughout the native and acellular esophagus, ureter and skin samples (Fig 6). Overall, collagen was more condensed in the acellular compared to the native samples, especially in the tunica muscularis of the esophagi (Fig 6B) and ureters (Fig 6D) as well as the subepidermal regions of the cutis (Fig 6F). In the esophagi and ureters, elastic fibers were observed especially in the submucosal region, the tunica muscularis and to some extent also in the adventitia and in blood vessels (Fig 7A–7D). The skin samples showed low quantities of elastic fibers, mostly in the vessels and dermal perivascular regions (Fig 7E and 7F). Similarly, fibronectin was present throughout the native and acellular esophagi and ureters (Fig 8A–8D), mainly located in the mucosal and muscular layers. The skin revealed lower contents of fibronectin, mostly situated in the epidermis and to a lesser extent in the dermis (Fig 8E). Acellularization of the skin was accompanied by a marked fibronectin washout in the dermis (Fig 8F).

### Electron microscopy revealed clotted type I collagens and loss of cross-linking fibers but intact collagen fibril structure

**Fig 9:**
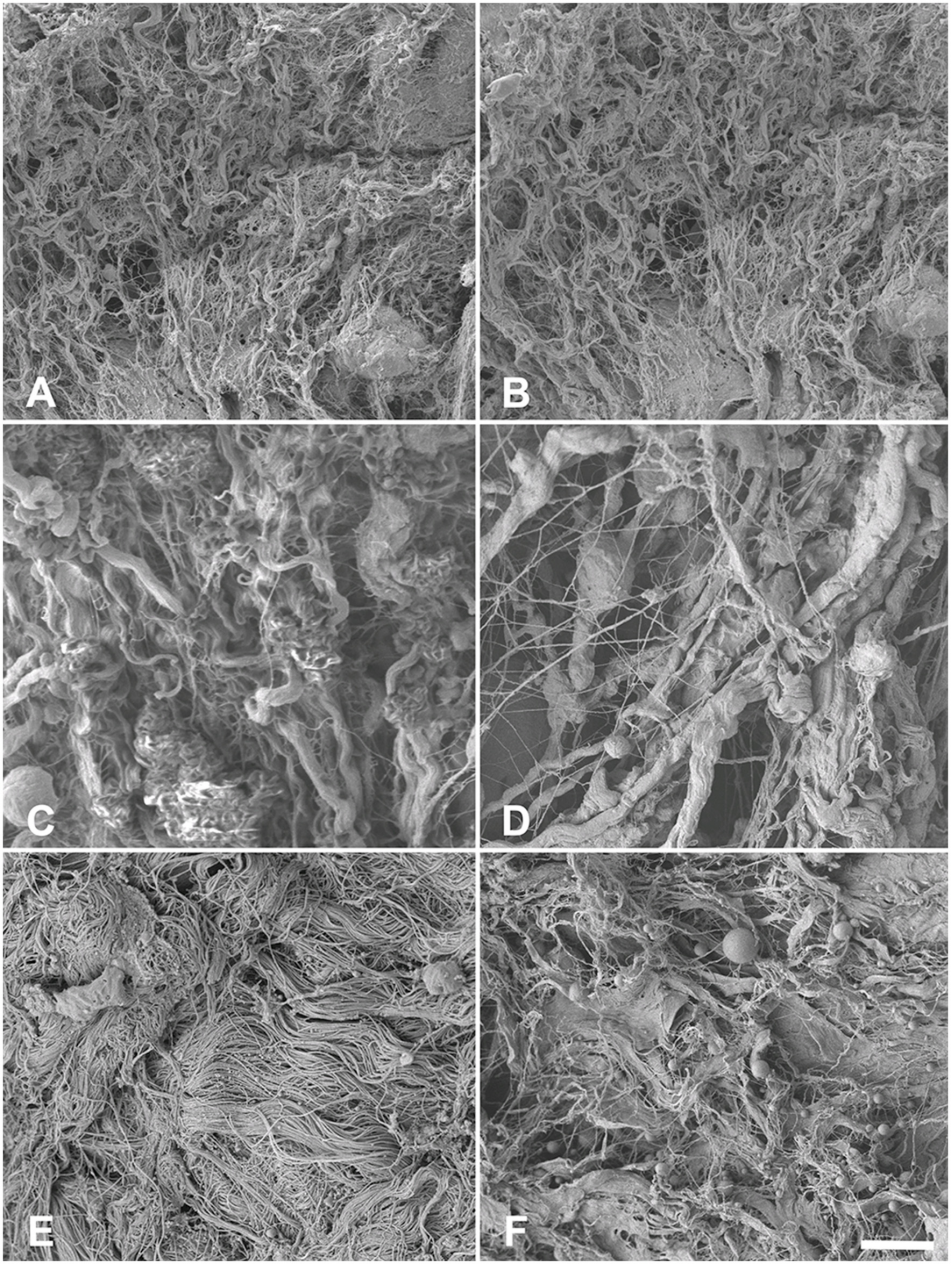
Scanning electron microscopy of 9A) native esophagus 9B) acellular esophagus 9C) native ureter 9D) acellular ureter 9E) native skin 9F) acellular skin. Type I collagens appeared remain largely intact in the 1000x magnification without noticeable major direction in the tunica muscularis of the esophagi and the ureters but in the dermal skin layer. Scale bar 10 μm.

**Fig 10:**
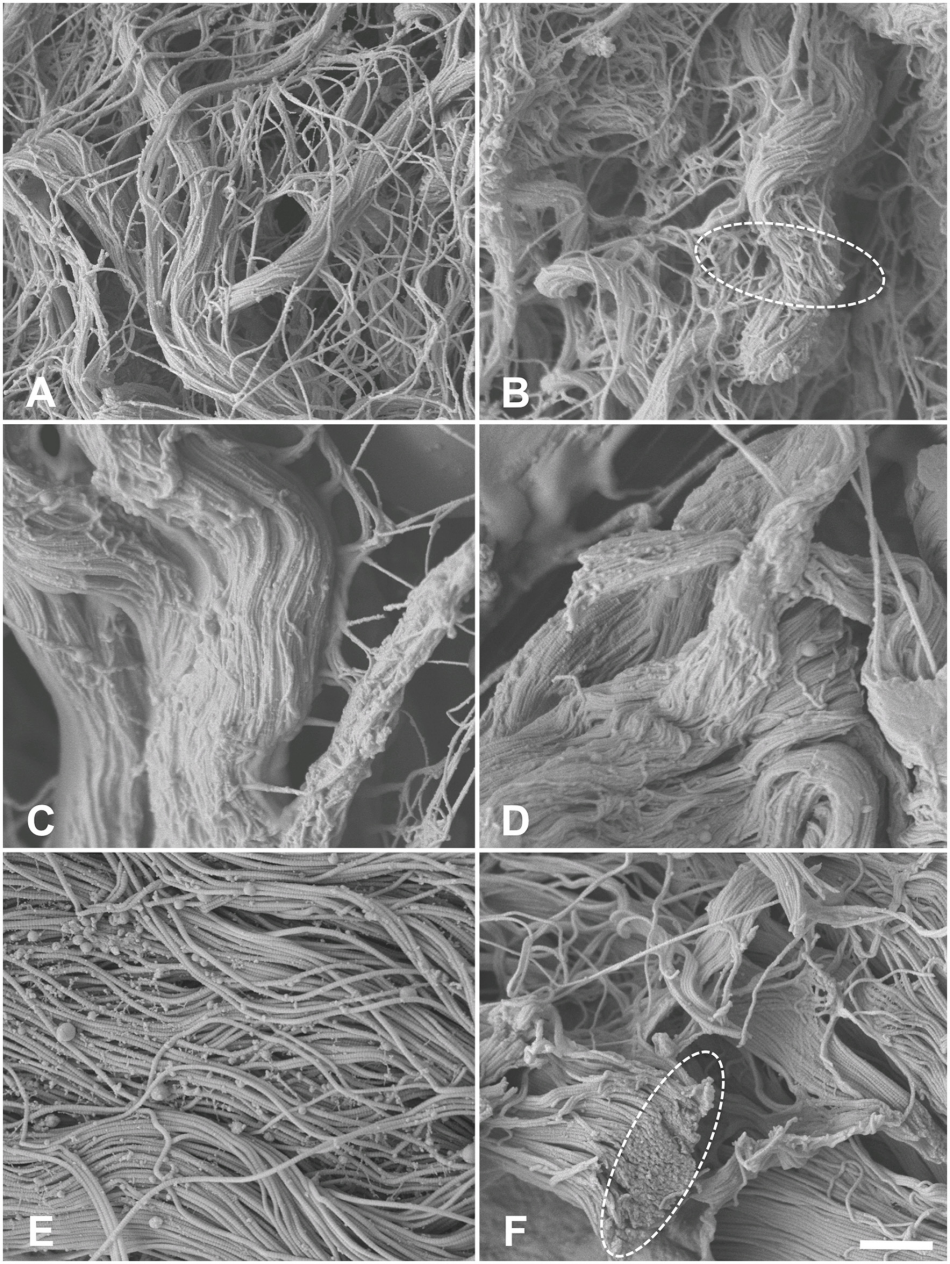
Scanning electron microscopy of 10A) native esophagus 10B) acellular esophagus 10C) native ureter 10D) acellular ureter 10E) native skin 10F) acellular skin. In the higher 10,000x magnification type I collagen clotting was observed (interrupted circles) accompanied by a loss of cross-linking collagens. Scale bar 1 μm.

**Fig 11:**
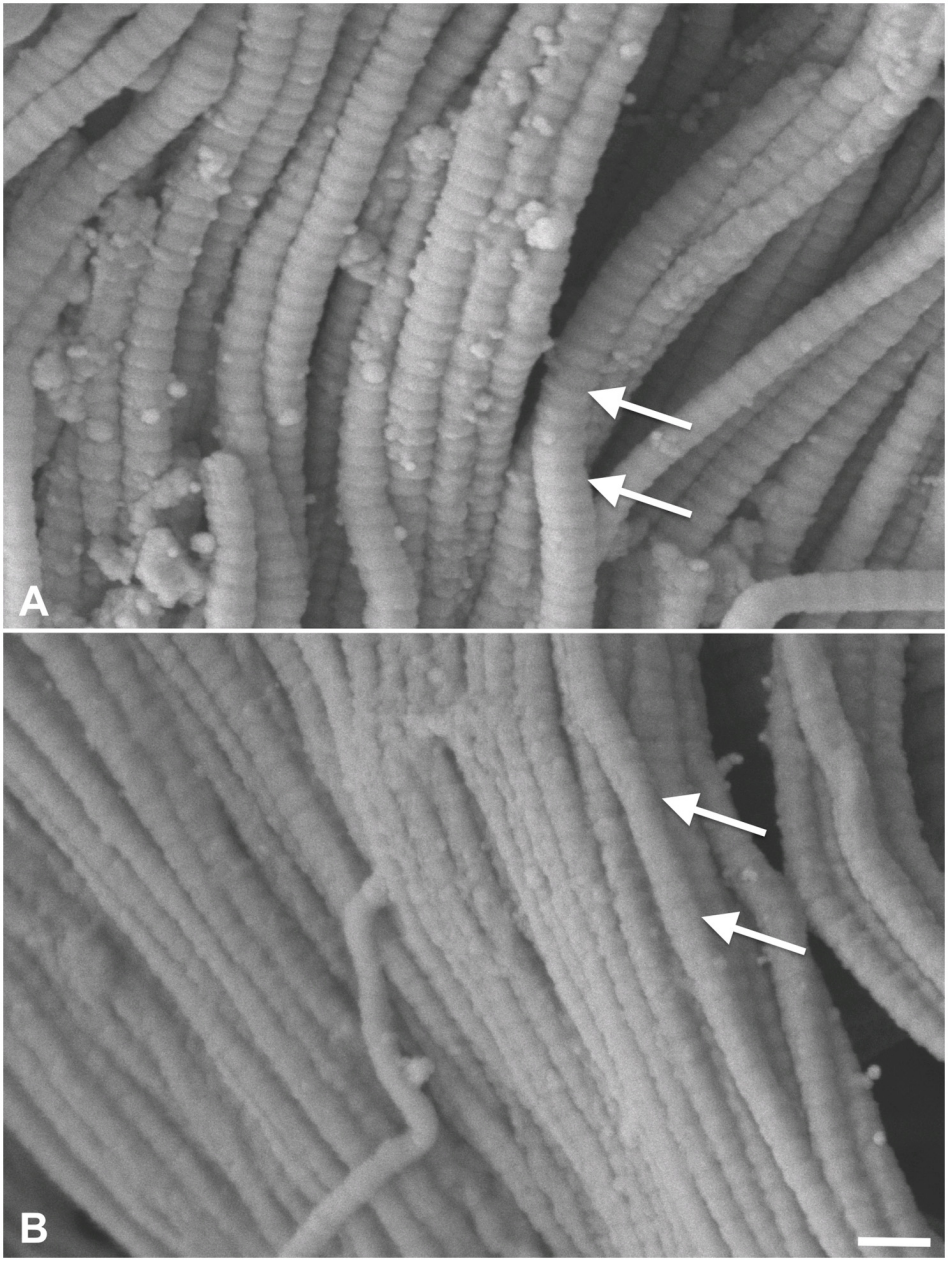
Scanning electron microscopy of the dermal areas of 11A) native skin 11B) acellular skin. Intact fibrils were observed at the 50,000x-magnification with the collagen-characteristic D-period at a 67-nm distance (white arrows), resembling the findings from transmission electron microscopy. Scale bar 150 nm.

**Fig 12:**
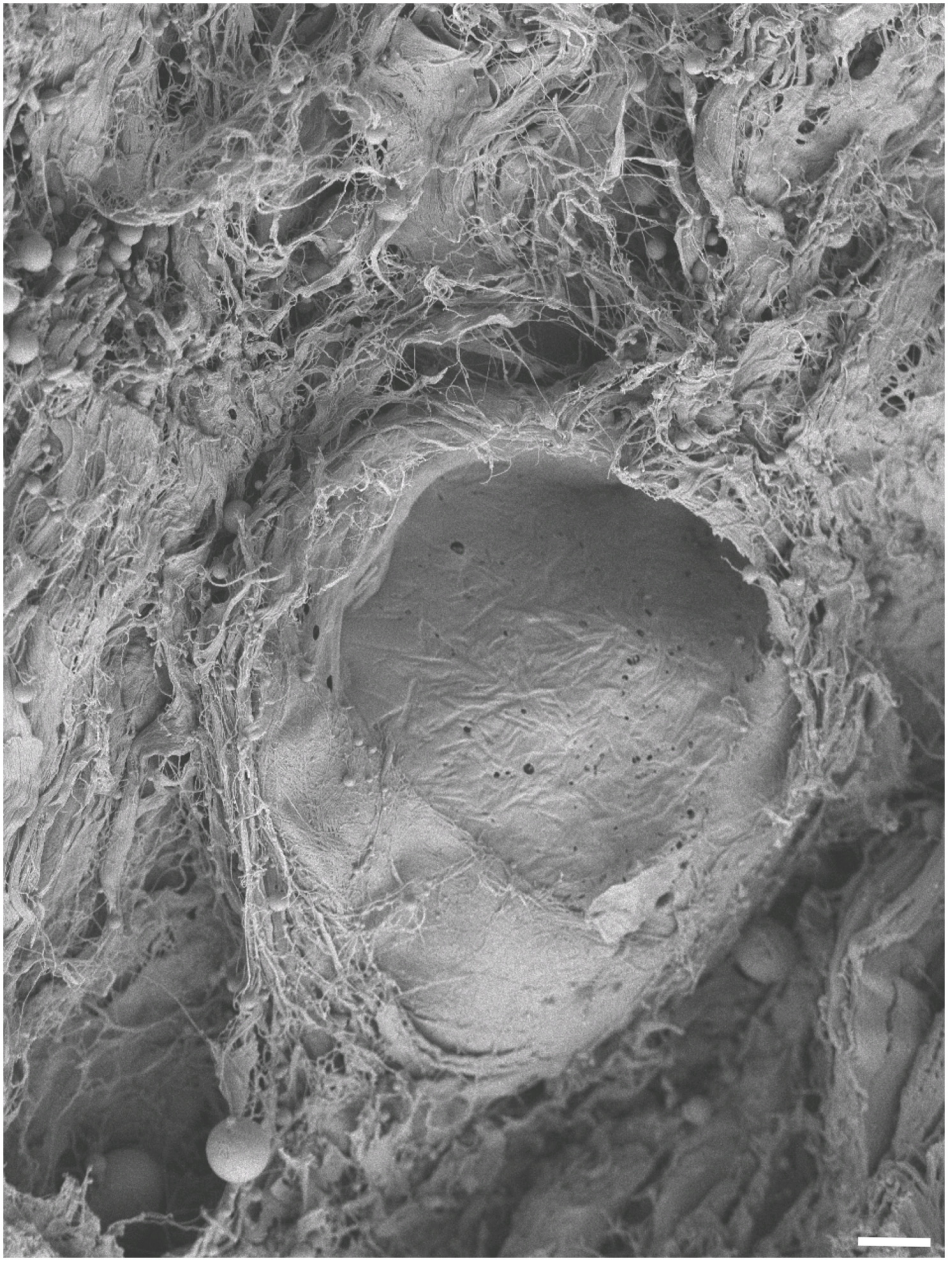
An emptied hair follicle root sheath is depicted in the subepidermal region of porcine skin. The surrounding tissue appears to be loosened and may be indicative of notching effects in skin samples. Scale bar 5 μm (1000x magnification).

In SEM, type I collagen fibers were found in high density (Fig 9). In the native esophagi, no major direction of fiber alignment could be observed (Fig 9A). Acellularization caused clotting of the collagen network (Fig 9B), which becomes more evident on the fibrillar level, accompanied by the loss of cross-linking fibers (Fig 10A and 10B). Similar observations were seen in tunica muscularis of the ureters (Figs 9C, 9D, 10C and 10D) and the skin (Fig 9E and 9F). Here, clotting appeared to be more pronounced (Fig 10E and 10F). Fiber and fibril directions were clearly distinguishable especially in the dermal regions (Figs 10E, 10F and 11). Emptied hair follicles and adjacent root sheaths were seen in the subepidermal layers of the skin (Fig 12). Here, the surrounding tissues appeared to be loosened.

**Fig 13:**
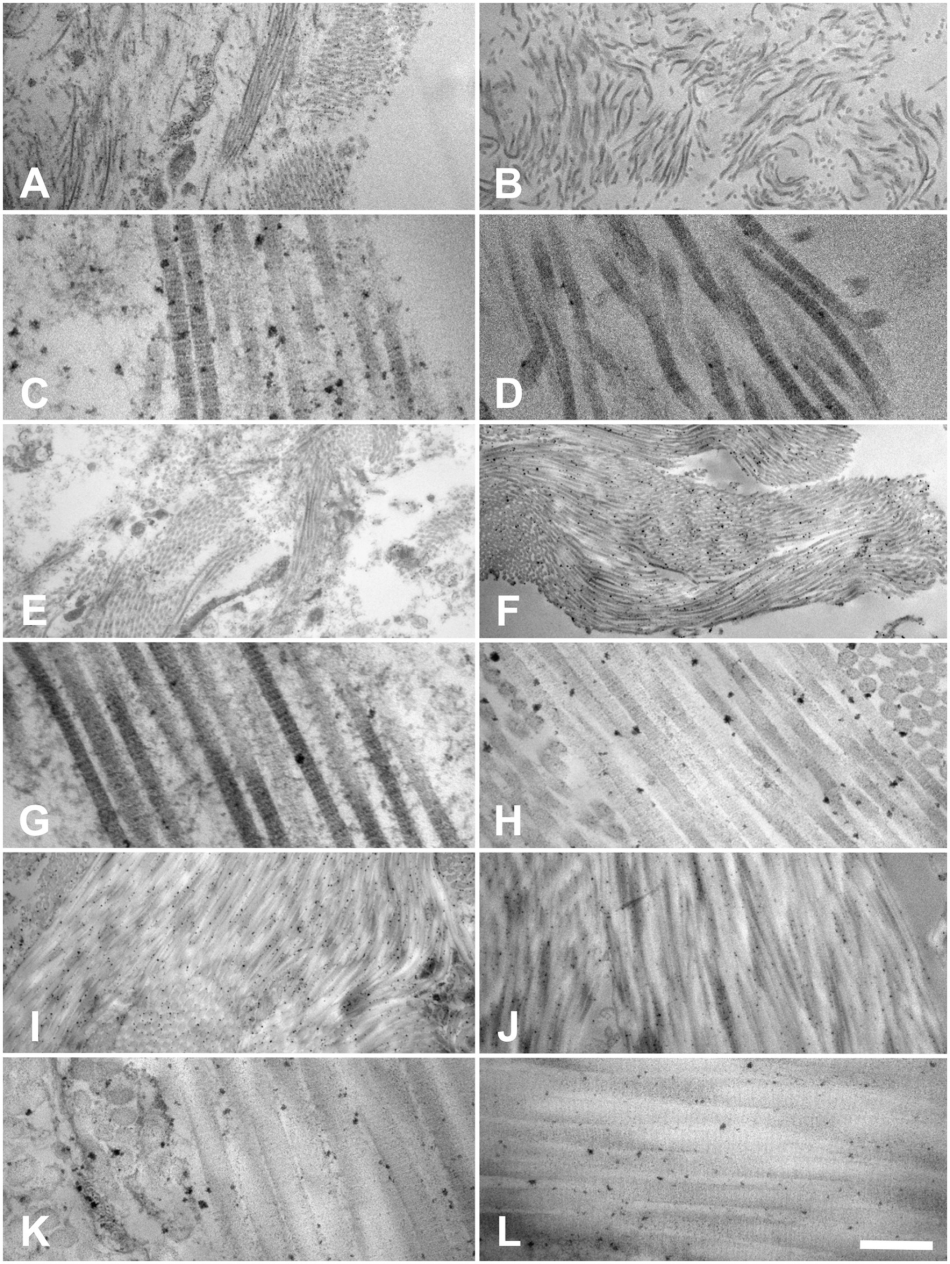
Transmission electron microscopy of 13A-D esophagi (tunica muscularis), 13E-H ureters (tunica muscularis), 13I-L skin (dermis) with focus on type I collagens. Left column (13A,C,E,G,I,K): native condition, right column (13B,D,F,H,J,L): acellular condition. Intact collagen fibers and fibrils were observed throughout the native and acellular scaffolds with the collagen-characteristic D-period at a 67-nm distance. Scale bar 1250 nm (13A,B,E,F,I,J; magnification 9700x) and 200 nm (13C,D,G,H,K,L; magnification 66,000x).

TEM confirmed that the collagen fibrils remained intact in both native and acellular samples (Fig 13). Fibril diameters were smaller in the esophagi and ureters compared to the skin. The characteristic 67-nm band was observed in both native and acellular samples (Fig 13), indicating structurally intact fibrils. No marked collagen fibril breakage was observed (Fig 11).

## Discussion

### Optimized test protocols facilitated direct comparison of the tensile properties of various soft tissues in native and the acellular conditions

One major aim of this study was to further optimize a setup for uniaxial tensile testing of soft tissues [23,25,26]. For this purpose, an osmotic stress protocol was established for each of the tissue types to adjust their water content close to the native situation. Furthermore, the samples’ ends were plastinated partially to minimize material slippage occurring during the tensile experiments [26] before the samples’ water content was adjusted for each group based on the osmotic stress protocol. Additionally, the sample dimensions were standardized in length and width during and after the plastination, using a template adapted from the German standard DIN 50125 [27]. Uniaxial tensile data were obtained from all samples using the same test parameters.

Additionally, samples’ cross sections were casted after preconditioning and before the tensile testing to further minimize the error introduced by different sample thickness. Finally, the native and acellular samples were compared successfully. Though a few samples were excluded from further evaluation because of the strict to inclusion criteria, this approach allowed for meaningful and comparable results.

### Reduced cross-sections indicated different cell quantities in the soft tissues

Acellularization-related reductions in the samples’ cross-sections varied depending on the origin of the tissues. In the esophagus samples, the significant reduction of 55% of the cross-section indicated that a vast majority of the tissue consists mainly of cells and only to a minority of ECM. This finding was confirmed by histology, where the different smooth muscle layers could hardly be differentiated in the acellular condition. In ureter samples the reduction of the cross section reached 32%, which was in line with the smaller dimensions of the samples and smooth muscle layers compared to the esophagus. In the skin, the reduction only reached 5%, demonstrating the it is a ECM-rich tissue.

### Acellularization increased elastic moduli and ultimate tensile stress in hollow organs, but decreased these values in skin

Acellularization induced increased elastic modulus and ultimate tensile stress values of hollow organs such as the esophagus and ureter. This may partially be attributed to the reductions in the samples cross sections in the acellular esophagi and ureters but also to the removal of muscle cells, which potentially have lower elastic moduli and ultimate tensile stress values in a non-contracted state compared to the ECM. However, the observed increase in the ultimate force values of the esophagus samples indicates that additional phenomena will likely be responsible for the increased load-bearing capacity following acellularization. These findings may likely be related to the ECM, although the total collagen, elastin and fibronectin contents appeared to be constant, as indicated by immunohistochemistry in previous experiments [21,22]. Collagen fibril structure remained intact. These findings are in line with our previous data on the nano-structural properties of human iliotibial tract collagens, using ^13^C CP MAS NMR [28].

From a functional point of view, the ECM serves as a backbone for the muscle cells forming the outer shape of the organs. The peristaltic function of these organs is guaranteed by contractile elements such as actin-myosin chains and which are a part of the intracellular matrix. Given the extensive networking between the muscle cells and the ECM, transverse forces occurring in the tensile experiments may weaken the composite structure in the native condition. Since the cells were largely removed, transverse forces may have largely been minimized to the effect of higher elastic moduli and ultimate tensile stress values in the acellular samples of esophagus and ureter. We observed ultrastructural evidence for altered collagen geometry in acellular esophagi and ureters. Collagen was clotted, accompanied by the loss of cross-linking fibers, potentially with a similar effect on decreasing transverse forces and increasing load-bearing capacity. This proposed reduction of transverse forces related to loss of cells and collagen crosslinking might be more pronounced in the acellular esophagi compared to the acellular ureters due to the relatively large tunica muscularis.

In contrast, the acellular skin samples yielded largely decreased elastic moduli and ultimate tensile stress values, accompanied by vast decreases in ultimate force. The tensile data are in line with previously published data on acellular skin [29]. Given the mechanical function of the skin to resist forces from various directions, the ECM might be regarded as a main mechanical actor of this organ. The dense network of elastins and collagens is interrupted by hair follicles and excretory ducts of sebaceous and sweat glands. Here, the primary function of cells may be production of ECM rather than primarily acting as a mechanical stabilizer. We suggest three reasons for the decreased load-bearing capacity of skin following acellularization. First, due to the acellularization procedure, the stratum corneum was completely removed, decreasing mechanical strength. Second, the removal of the hair follicles may have caused the potential weakening of the skin. In the native condition, the follicles filled spaces as incompressible elements. In the acellular condition, the SDS washed out the follicles, which left behind cavities, which may cause notch effects in the uniaxial tensile experiments, further weakening the skin. We found ultrastructural evidence supporting this theory with loosened collagen networks surrounding the emptied follicles, but intact collagen fibrils. Third, the loss of proteoglycans with adjacent fibronectin may have caused inferior tensile properties. Fibronectin is a binding protein of the formed ECM fraction. Proteoglycans are well-known to be altered by SDS [4,30]. In our given setup, skin acellularization needed 28 days, but only 7 days for the esophagus and ureter specimens. This comparably long time frame may have facilitated the proteoglycan washout, lowering mechanical strength.

Acellularization-related changes in maximum strain were minute for esophagi, ureters and skin. This finding indicates that the passive properties of the respective cells hardly influence the tensile properties of the tissues in terms of mechanical strain. Thus, the tensile mechanical properties were altered tissue-dependently, to a varying extent and differently for the different tissues, indicating that the influence of the cellular components and/or the chemicals used for acellularization is different. Hypothesis 1, claiming that tensile properties of native or acellular porcine samples are different in esophagi, ureters and skin, can therefore be accepted.

### Clinical implications

Acellular scaffolds are frequently used for the surgical reconstruction of tendon, ligament or other soft tissue injuries [1,4,15,31–35]. Vascular and cardiac surgery is another field of application [4,36–38]. Another potential application would be the surgical reconstruction of the esophagus following burns. Additionally, esophageal tumors or tumors of genitourinary tract are potential sites of application. The main advantage of acellular scaffolds is their reduced antigenicity due to the partial or complete absence of cellular proteins [30]. Short repopulation rates were shown elsewhere for the esophagus [22] and ureters [21]. However, the acellularization might be accompanied by altered mechanical properties and a potential weakening of the scaffolds, which are intended to be implanted [4,39]. The same is true for repopulated scaffolds, in which degradation processes were found following implantation [15,39]. Our direct comparison of native versus acellular tissue also indicated that alterations in the mechanical properties may not only be caused by degradation of ECM in the scaffolds, but also by the load-altering properties of cells.

An in-depth knowledge on scaffold behavior is of interest not only for orthotopic but also for heterotopic implantations. Inter comparison of the esophagus, ureter and skin samples showed that each of the tissues had different mechanical properties in both the native and acelluar condition. Especially the decrease in the elastic modulus and ultimate tensile stress of skin samples is an indicator of the difference in behavior of the esophagi and ureters. Hypothesis 2, claiming that acellularization causes changes in the tensile properties of esophagi, ureters and skin, as compared to the native condition, can therefore also be accepted. Consequently, from a biomechanical point of view, acellular esophagus and ureter samples may be recommended as scaffolds for the orthotopic surgical reconstruction or replacement. This is not the case for acellular skin scaffolds treated with SDS, if the dermal scaffolds are likely to be subjected to significant mechanical loads.

The third hypothesis, stating that changes in the tensile properties of acellular scaffolds are accompanied by morphological alteration in the scaffolds, can likewise be accepted. Beyond extensive cell removal, we observed collagen clotting and loss of cross-linking collagen fibers in acellular esophagus and ureter samples causing increased mechanical tensile strength. *Vice versa*, we found a fibronectin removal in acellular skin samples having decreased mechanical strength.

### Limitations

First, only a limited number of suitable samples was available for the study, which was partially related to our strict inclusion criteria. Second, using SDS resulted in a different duration of acellularization to the effect that degradation may have influenced the values to a different extent. Third, the effects of cross linking scaffolds by various chemicals have not been investigated in this study. Further experiments would help substantiate the effects of different acellularization protocols on the effectiveness and the mechanical properties not only *in vitro* as in our study, but also *in vivo* in long-term experiments.

## References

[1] Chen J, Xu J, Wang A, Zheng M (2009) Scaffolds for tendon and ligament repair: review of the efficacy of commercial products. Expert Rev Med Devices 6 (1): 61–73. 10.1586/17434440.6.1.61 19105781

[2] Pridgen BC, Woon CYL, Kim M, Thorfinn J, Lindsey D, Pham H et al (2011) Flexor tendon tissue engineering: acellularization of human flexor tendons with preservation of biomechanical properties and biocompatibility. Tissue Engineering Part C: Methods 17 (8): 819–828. 21548795 10.1089/ten.tec.2010.0457

[3] Rubin L, Schweitzer S (2005) The use of acellular biologic tissue patches in foot and ankle surgery. Clin Podiatr Med Surg 22 (4): 533–52, vi 16213378 10.1016/j.cpm.2005.08.002

[4] Gilbert TW, Sellaro TL, Badylak SF (2006) Decellularization of tissues and organs. Biomaterials 27 (19): 3675–3683. 16519932 10.1016/j.biomaterials.2006.02.014

[5] Badylak SF, Freytes DO, Gilbert TW (2009) Extracellular matrix as a biological scaffold material: Structure and function. Acta Biomaterialia 5 (1): 1–13. 10.1016/j.actbio.2008.09.013 18938117

[6] Crapo PM, Gilbert TW, Badylak SF (2011) An overview of tissue and whole organ decellularization processes. Biomaterials 32 (12): 3233–3243. 10.1016/j.biomaterials.2011.01.057 21296410 PMC3084613

[7] Song JJ, Ott HC (2011) Organ engineering based on decellularized matrix scaffolds. Trends in Molecular Medicine 17 (8): 424–432. 10.1016/j.molmed.2011.03.005 21514224

[8] Sundermann SH, Muggler O, Caliskan E, Reser D, Manka R, Holubec T et al (2015) Extracellular matrix for reconstruction of cardiac structures after tumour resections. Interact Cardiovasc Thorac Surg 20 (1): 10–14. 10.1093/icvts/ivu310 25232129

[9] Rovak JM, Bishop DK, Boxer LK, Wood SC, Mungara AK, Cederna PS (2005) Peripheral nerve transplantation: the role of chemical acellularization in eliminating allograft antigenicity. Journal of reconstructive microsurgery 21 (3): 207–213. 15880301 10.1055/s-2005-869828

[10] Wainwright D, Madden M, Luterman A, Hunt J, Monafo W, Heimbach D et al (1996) Clinical evaluation of an acellular allograft dermal matrix in full-thickness burns. Journal of Burn Care & Research 17 (2): 124–136. 10.1097/00004630-199603000-00006 8675502

[11] Schmidt CE, Baier JM (2000) Acellular vascular tissues: natural biomaterials for tissue repair and tissue engineering. Biomaterials 21 (22): 2215–2231. 11026628 10.1016/s0142-9612(00)00148-4

[12] Cebotari S, Mertsching H, Kallenbach K, Kostin S, Repin O, Batrinac A et al (2002) Construction of autologous human heart valves based on an acellular allograft matrix. Circulation 106 (12 suppl 1): I. 12354711

[13] Jo W, Sohn Y, Choi YH, Kim HJ, Cho HD (2007) Modified acellularization for successful vascular xenotransplantation. Journal of Korean medical science 22 (2): 262–269. 17449935 10.3346/jkms.2007.22.2.262 PMC2693593

[14] Sung H, Liang H (2003) Repair of ligaments, tendons, muscle and cartilage using a graft formed of connective tissue crosslinked with genipin: Google Patents.

[15] Adams JE, Zobitz ME, Reach JS, An K, Steinmann SP (2006) Rotator cuff repair using an acellular dermal matrix graft: an in vivo study in a canine model. Arthroscopy: The Journal of Arthroscopic & Related Surgery 22 (7): 700–709. 16843804 10.1016/j.arthro.2006.03.016

[16] Branch JP (2011) A tendon graft weave using an acellular dermal matrix for repair of the Achilles tendon and other foot and ankle tendons. J Foot Ankle Surg 50 (2): 257–265. 10.1053/j.jfas.2010.12.015 21354014

[17] Ehsan A, Lee DG, Bakker AJ, Huang JI (2012) Scapholunate ligament reconstruction using an acellular dermal matrix: a mechanical study. The Journal of hand surgery 37 (8): 1538–1542. 10.1016/j.jhsa.2012.04.043 22749483

[18] Borschel GH, Kia KF, Kuzon WM Jr, Dennis RG (2003) Mechanical properties of acellular peripheral nerve. Journal of Surgical Research 114 (2): 133–139. 14559438 10.1016/s0022-4804(03)00255-5

[19] Hopper RA, Woodhouse K, Semple JL (2003) Acellularization of Human Placenta With Preservation of the Basement Membrane: A Potential Matrix for Tissue Engineering. Annals of Plastic Surgery 51 (6). 10.1097/01.sap.0000095658.46675.76 14646657

[20] Brown AL, Farhat W, Merguerian PA, Wilson GJ, Khoury AE, Woodhouse KA (2002) 22 week assessment of bladder acellular matrix as a bladder augmentation material in a porcine model. Biomaterials 23 (10): 2179–2190. 11962659 10.1016/s0142-9612(01)00350-7

[21] Koch H, Hammer N, Ossmann S, Schierle K, Sack U, Hofmann J et al (2015) Tissue engineering of ureteral grafts: preparation of biocompatible crosslinked ureteral scaffolds of porcine origin. Frontiers in bioengineering and biotechnology 3. 10.3389/fbioe.2015.00089 PMC4477215 26157796

[22] Koch H, Graneist C, Emmrich F, Till H, Metzger R, Aupperle H et al (2012) Xenogenic Esophagus Scaffolds Fixed with Several Agents: Comparative In Vivo Study of Rejection and Inflammation. Journal of Biomedicine and Biotechnology 2012: 11. 10.1155/2012/948320 PMC3312382 22505820

[23] Hammer N, Lingslebe U, Aust G, Milani TL, Hädrich C, Steinke H (2012) Ultimate stress and age-dependent deformation characteristics of the iliotibial tract. Journal of the mechanical behavior of biomedical materials 16: 81–86. 10.1016/j.jmbbm.2012.04.025 23178479

[24] Steinke H, Lingslebe U, Böhme J, Slowik V, Shim V, Hädrich C et al (2012) Deformation behavior of the iliotibial tract under different states of fixation. Medical engineering & physics 34 (9): 1221–1227. 22297087 10.1016/j.medengphy.2011.12.009

[25] Hammer N, Huster D, Fritsch S, Hädrich C, Koch H, Schmidt P et al (2014) Do cells contribute to tendon and ligament biomechanics. PLoS ONE 9 (8): e105037 EP -. 10.1371/journal.pone.0105037 25126746 PMC4134275

[26] Sichting F, Steinke H, Wagner MF, Fritsch S, Hädrich C et al (2015) Quantification of material slippage in the iliotibial tract when applying the partial plastination clamping technique. Journal of the mechanical behavior of biomedical materials 49: 112–117. 10.1016/j.jmbbm.2015.04.028 26005842

[27] Deutsches Institut für Normung e.V.: Prüfung metallischer Werkstoffe (2009) Prüfung metallischer Werkstoffe -Zugproben (DIN 50125 77.040.10). Berlin: Beuth Verlag GmbH.

[28] Hammer N, Huster D, Boldt A, Hädrich C, Koch H, Hammer N (2016) A preliminary technical study on sodium dodecyl sulfate-induced changes of the nano-structural and macro-mechanical properties in human iliotibial tract specimens. Journal of the mechanical behavior of biomedical materials. 10.1016/j.jmbbm.2016.01.018 26866452

[29] Hoganson DM, O’Doherty EM, Owens GE, Harilal DO, Goldman SM, Bowley CM et al (2010) The retention of extracellular matrix proteins and angiogenic and mitogenic cytokines in a decellularized porcine dermis. Biomaterials 31 (26): 6730–6737. 10.1016/j.biomaterials.2010.05.019 20576289

[30] Schulze-Tanzil G, Al-Sadi O, Ertel W, Lohan A (2012) Decellularized Tendon Extracellular Matrix—A Valuable Approach for Tendon Reconstruction. Cells 1 (4): 1010–1028. 10.3390/cells1041010 24710540 PMC3901141

[31] Lee DK (2008) A preliminary study on the effects of acellular tissue graft augmentation in acute Achilles tendon ruptures. The Journal of Foot and Ankle Surgery 47 (1): 8–12. 18156058 10.1053/j.jfas.2007.08.015

[32] Barber FA, Aziz-Jacobo J (2009) Biomechanical testing of commercially available soft-tissue augmentation materials. Arthroscopy: The Journal of Arthroscopic & Related Surgery 25 (11): 1233–1239. 19896044 10.1016/j.arthro.2009.05.012

[33] Ellis CV, Kulber DA (2012) Acellular dermal matrices in hand reconstruction. Plastic and reconstructive surgery 130 (5S-2): 256S. 23096981 10.1097/PRS.0b013e318265a5cf

[34] Rao BM, Kamal TT, Vafaye J, Taylor L (2012) Surgical repair of hip abductors. A new technique using Graft Jacket^®^ allograft acellular human dermal matrix. International orthopaedics 36 (10): 2049–2053. 10.1007/s00264-012-1630-6 22872412 PMC3460093

[35] Lovati AB, Bottagisio M, Moretti M (2016) Decellularized and Engineered Tendons as Biological Substitutes: A Critical Review. Stem Cells International 2016. 10.1155/2016/7276150 PMC4736572 26880985

[36] Chung S, Hazen A, Levine JP, Baux G, Olivier WA, Yee HT et al (2003) Vascularized acellular dermal matrix island flaps for the repair of abdominal muscle defects. Plastic and reconstructive surgery 111 (1): 225–232. 12496583 10.1097/01.PRS.0000034934.05304.ED

[37] Hülsmann J, Grün K, El Amouri S, Barth M, Hornung K, Holzfuß C et al (2012) Transplantation material bovine pericardium: biomechanical and immunogenic characteristics after decellularization vs. glutaraldehyde‐fixing. Xenotransplantation 19 (5): 286–297. 10.1111/j.1399-3089.2012.00719.x 22978462

[38] Byrom MJ, Ng MKC, Bannon PG (2013) Biomechanics and biocompatibility of the perfect conduit—can we build one. Annals of Cardiothoracic Surgery 2 (4): 435 10.3978/j.issn.2225-319X.2013.05.04 23977620 PMC3741889

[39] Tischer T, Aryee S, Wexel G, Steinhauser E, Adamczyk C, Eichhorn S et al (2009) Tissue engineering of the anterior cruciate ligament—sodium dodecyl sulfate-acellularized and revitalized tendons are inferior to native tendons. Tissue Engineering Part A 16 (3): 1031–1040. 10.1089/ten.TEA.2009.0043 19845462

